# Supplementary material for: Data-driven cultural background fusion for environmental art image classification: Technical support of the dual Kernel squeeze and excitation network
Source: PLoS One. 2025 Mar 20;20(3):e0313946. doi: 10.1371/journal.pone.0313946 (PMC11925309; doi:10.1371/journal.pone.0313946)
Supplement: S1 Data — (ZIP) [file pone.0313946.s001.zip › ╩2╛▌░n/code description.docx]

**Code description:** The code provides the complete implementation code of the Dual Kernel Squeeze and Excitation Network (DKSE-Net), which is constructed based on the PyTorch framework. First, the core operations in the Squeeze-and-Excitation (SENet) module are defined. The module generates the global statistics information of the feature map by Global Average Pooling (GAP) operation and processes the information through two fully connected layers to obtain the channel weight of the feature map. These weights are applied to the input feature map using the Sigmoid activation function to enhance the features of the important channels. Next, the code implements the dilated convolution module. Dilated convolution expands the receptive field by inserting intervals (dilation) between the convolution kernel without increasing the computational effort or losing spatial resolution. This operation is essential for capturing contextual information in artistic images with complex cultural contexts. Then, a pointwise convolution module is used for linear combination and dimensionality reduction of the channels in the feature graph, which effectively reduces the computational complexity while maintaining the relationships between channels. The DKSE module is implemented in code through the DKSEBlock category. The module includes multiple convolution operations, dilated convolution, SENet operations, pointwise convolution, and Dropout technology. The Dropout technology randomly drops the activation values of some neurons during training to prevent overfitting of the model, thereby improving the model's generalization ability. In the DKSEBlock, the feature maps are processed by two convolution kernels of different sizes and then weighted by the SENet module. Finally, a pointwise convolution operation is performed, and the robustness of the model is enhanced by Dropout technology. The DKSENet category implements the complete network model structure. The network first passes through a standard convolution layer and batch normalization (BN) layer, then enters the DKSE module for feature extraction, and finally through the fully connected layer for classification. The model’s input is the art image, and its output is the prediction probability of each category. During the training, the Adam Optimizer and the Cross-Entropy Loss function are used to optimize the model. In the training loop, the code shows how to train and validate the model. In each training epoch, the model calculates the loss through forward propagation and then updates the model parameters through backpropagation. After the training is completed, the model's performance on the test set is evaluated to calculate classification accuracy. Notably, the code data loaders (`train_loader` and `test_loader`) are configured correctly and contain data for training and validation. Users need to adapt the data loading and preprocessing steps according to the specific dataset and task requirements to ensure that the model can perform the expected performance in practical applications.
